# Results of a Dose‐Finding Phase 1b Study of Subcutaneous Atezolizumab in Patients With Locally Advanced or Metastatic Non–Small Cell Lung Cancer

**DOI:** 10.1002/cpdd.936

**Published:** 2021-03-31

**Authors:** Enriqueta Felip, Mauricio Burotto, Zanete Zvirbule, Luis A. Herraez‐Baranda, Pascal Chanu, Smita Kshirsagar, Vidya Maiya, Phyllis Chan, Emanuela Pozzi, Mathilde Marchand, Marion Monchalin, Kunihiko Tanaka, Nadia Tosti, Bei Wang, Eleonora Restuccia

**Affiliations:** ^1^ Vall d'Hebron University Hospital and Institute of Oncology (VHIO) UVic‐UCC, IOB‐Quiron Barcelona Spain; ^2^ Centro de Investigación Clínica Bradford Hill Santiago Chile; ^3^ Riga Eastern Clinical University Hospital Riga Latvia; ^4^ F. Hoffmann‐La Roche, Ltd. Basel Switzerland; ^5^ Genentech/Roche Lyon France; ^6^ Genentech, Inc. South San Francisco California USA; ^7^ Certara Strategic Consulting Paris France

**Keywords:** atezolizumab, hyaluronanidase, NSCLC, pharmacokinetics, subcutaneous

## Abstract

Intravenous (IV) atezolizumab is approved for non–small cell lung and other cancers. Subcutaneous (SC) atezolizumab coformulated with recombinant human hyaluronidase, a permeation enhancer for SC dispersion and absorption, is being developed to improve treatment options, reduce burden, and increase efficiency for patients and practitioners. IMscin001 (NCT03735121), a 2‐part, open‐label, global, multicenter, phase 1b/3 study, is evaluating the pharmacokinetics (PK), safety, and efficacy of SC atezolizumab. The part 1 (phase 1b) objective was determination of an SC atezolizumab dose yielding a serum trough concentration (C_trough_) comparable with IV. Patients enrolled in 3 cohorts received SC atezolizumab 1800 mg (thigh) once (cohort 1), 1200 mg (thigh) every 2 weeks for 3 cycles (cohort 2), or 1800 mg (abdomen) every 3 weeks cycle 1, then cycles 2 and 3 (thigh) every 3 weeks (cohort 3). In subsequent cycles, IV atezolizumab 1200 mg every 3 weeks was administered until loss of clinical benefit. SC atezolizumab 1800 mg every 3 weeks and 1200 mg every 2 weeks provided similar C_trough_ and area under the curve values in cycle 1 to the corresponding IV atezolizumab reference, was well tolerated, and exhibited a safety profile consistent with the established IV formulation. Exposure following SC injection in the abdomen was lower (20%, 28%, and 27% for C_trough_, maximum concentration, and area under the concentration‐time curve from time 0 to day 21, respectively) than in the thigh. Part 1 SC and IV PK data were analyzed using a population PK modeling approach, followed by simulations. Part 2 (phase 3) will now be initiated to demonstrate that SC atezolizumab PK exposure is not lower than that of IV.

Atezolizumab (Tecentriq; F. Hoffmann‐La Roche Ltd, Basel, Switzerland), a programmed death‐ligand 1 immune checkpoint inhibitor, has received approval by regulatory authorities in Europe, the United States, and many other countries as monotherapy or in combination with chemotherapy and/or targeted therapy for the treatment of patients with locally advanced or metastatic non–small cell lung cancer (NSCLC), small cell lung cancer, triple‐negative breast cancer, urothelial cancer, hepatocellular carcinoma, and unresectable or metastatic melanoma.^1^, ^2^, ^3^, ^4^, ^5^, ^6^, ^7^, ^8^, ^9^, ^10^ Currently, atezolizumab is available only as an intravenous (IV) formulation.

Selection of the IV atezolizumab 1200 mg every‐3‐week dosing regimen was informed by nonclinical studies identifying a minimum blood plasma concentration of 6 μg/mL, as well as clinical studies with 1200 mg every 3 weeks that achieved a minimum blood plasma concentration >6 μg/mL in >95% of patients in various indications, and as monotherapy or in combination with other agents.^11^, ^12^, ^13^, ^14^, ^15^ The pharmacokinetic (PK) profile was similar to that expected with an immunoglobulin G1 antibody, with a terminal half‐life of 27 days and steady‐state concentrations reached after 6 to 9 weeks.^12^, ^16^ Atezolizumab had linear PK over 1 to 20 mg/kg IV every 3 weeks. The clearance was 0.2 L/day, with central and peripheral distribution volumes estimated at 3.28  and 3.63 L, respectively; similar results were observed between adults and pediatric patients.^12^, ^17^ Further model‐based simulations demonstrated the interchangeable use of IV atezolizumab at 840 mg every 2 weeks, 1200 mg every 3 weeks, and 1680 mg every 4 weeks.^16^ No clinically meaningful exposure‐efficacy or exposure‐safety relationships were observed with IV atezolizumab.^12^, ^16^, ^17^

However, crossover studies with other monoclonal antibodies such as trastuzumab and rituximab have demonstrated that the majority of patients prefer the subcutaneous (SC) route of administration over IV.^18^, ^19^, ^20^, ^21^, ^22^ Preferences for SC have been related to prospects of spending less time in the clinic, less pain and discomfort, easier administration compared with IV infusion, and the possibility of receiving the injection in the home rather than the clinic.^18^, ^19^, ^20^, ^21^, ^22^, ^23^, ^24^ Moreover, many clinicians overwhelmed with the number of patients seen daily also prefer SC over IV administration for its potential to improve workflow efficiency and scheduling flexibility, minimal impact on resourcing, and facilitation of treatment of patients with fluid restrictions or difficult venous access.^25^, ^26^, ^27^ A potential outcome of a streamlined workflow with SC administration can be reductions in direct and indirect health care costs due to reduction in drug waste during preparation,^28^, ^29^, ^30^, ^31^, ^32^, ^33^, ^34^ shorter administration time, and fewer potential adverse events (AEs) associated with IV administration that may require additional management.^25^, ^35^

Because reconstituted monoclonal antibodies often require a large volume of solution for dissolution, pain‐free administration of large‐volume SC injections can be challenging.^35^ Hyaluronan (hyaluronic acid), a large, endogenous glycosaminoglycan in the skin and underlying SC tissue, combines with water in the tissue to create a barrier in the form of a gel‐like substance. As a result, the delivery, distribution, and absorption of medications administered SC can be limited.^35^, ^36^ Recombinant human hyaluronidase PH20 (rHuPH20, ENHANZE drug delivery technology; Halozyme, Inc, San Diego, California) is an endoglycosidase that transiently degrades hyaluronan at the SC injection site, resulting in enhanced tissue permeability and improved dispersion and absorption of large‐volume, coadministered drugs, thereby acting as a bridge between IV and SC modalities.^36^ The administration of monoclonal antibodies by the SC route has become an established treatment modality for the treatment of solid and hematologic tumors, resulting in several approvals in recent years for coformulations with rHuPH20 in the United States and Europe.^37^, ^38^, ^39^, ^40^, ^41^, ^42^, ^43^

Historically, clinical development programs for SC monoclonal antibodies have used a PK‐bridging approach for agents previously approved for IV use.^44^, ^45^ The underlying premise of this approach is that the selected SC dose under evaluation will achieve a serum trough concentration (C_trough_) that is at least as high as the serum C_trough_ of the previously approved IV formulation of the same drug. If successful, the SC formulation should achieve the same degree of receptor saturation and, therefore, the same efficacy as documented with the IV treatment. The identified SC dose is then evaluated for demonstration of noninferiority of PK exposure vs IV administration based on C_trough_ at cycle 1 (just before the next dose is administered in cycle 2).^44^, ^45^

Given this precedence and to establish the IV to SC bridge for atezolizumab, a 2‐part, phase 1b/3 study, IMscin001, is being conducted to help improve access, convenience, and treatment experience for patients, as well as facilitate cost efficiency and provide a streamlined workflow for health care practitioners. The goal of part 1 (phase 1b) of the study was to determine the dose of an SC formulation of atezolizumab that would yield exposure comparable with that of the IV formulation. The results of this study will inform further development of a ready‐to‐use SC atezolizumab in part 2, a phase 3 confirmatory PK study. The methodology and results of Part 1 are reported here.

## Methods

Approval for the protocol was obtained from the investigational review board or independent ethics committee at each study site (see Supplemental Information for listing of 16 study sites). For enrollment, patients must have signed the informed consent form and be ≥18 years of age.

IMscin001 (NCT03735121) is a 2‐part, open‐label, global, multicenter, phase 1b/3 study to evaluate the PK, safety, and efficacy of SC atezolizumab compared with IV atezolizumab in patients with locally advanced or metastatic NSCLC. This clinical study was sponsored by F. Hoffmann‐La Roche Ltd (Basel, Switzerland). It was conducted in full concordance with the International Council for Harmonisation E6 guideline for Good Clinical Practice and the principles of the Declaration of Helsinki.

### Study Objectives

Part 1 was designed to determine the dose of SC atezolizumab that provides a comparable C_trough_ to that following administration of IV atezolizumab 1200 mg administered once every 3 weeks. Another PK objective was characterization of the PK profile of SC atezolizumab on the basis of serum concentrations of atezolizumab at specified time points during SC administration. Safety objectives included the incidence and severity of AEs (determined by the National Cancer Institute Common Terminology Criteria for Adverse Events, version 5.0) as well as the incidence and severity of targeted vital signs and clinical laboratory abnormalities.

### Study Design

Part 1, the dose‐finding segment of this study, was composed of 3 cohorts for the evaluation of 2 different SC doses (ie, 1200 mg and 1800 mg), administration frequencies (ie, once, every 2 weeks, or every 3 weeks), and sites of injection (ie, thigh or abdomen) of atezolizumab (see Procedures). Enrollment of the first 6 patients in cohort 1 was staggered to create a 72‐hour interval for evaluation of the safety and tolerability of the study drug before the next patient was enrolled.

### Patients

Patients diagnosed with advanced or metastatic (ie, stage IIIB not eligible for definitive chemoradiotherapy, stage IV, or recurrent) NSCLC previously treated with platinum‐based chemotherapy were included in the study. Key inclusion criteria included presence of measurable disease as defined by Response Evaluation Criteria in Solid Tumors, version 1.1; Eastern Cooperative Oncology Group performance status of 0 or 1; life expectancy of ≥12 weeks; adequate hematologic and end‐organ function based on assessment of pertinent laboratory parameters; and normal intact skin at the injection site. Key exclusion criteria included prior cancer immunotherapy; systemic immunostimulatory or immunosuppressive agents; symptomatic, untreated, or actively progressing central nervous system metastases; uncontrolled or symptomatic hypercalcemia; active or history of autoimmune disease or immune deficiency; pulmonary fibrosis; pneumonia or pneumonitis; significant cardiovascular disease; history of malignancies other than NSCLC in the previous 5 years; and allergy or sensitivity to any ingredient of atezolizumab or rHuPH20.

### Procedures

The formulation and concentration of rHuPH20 used in this study was the same as that used in the SC formulations of trastuzumab, rituximab, the fixed‐dose coformulation of pertuzumab and trastuzumab, and daratumumab.^37^, ^38^, ^39^, ^40^, ^41^, ^42^, ^43^ In part 1 of this study, SC atezolizumab was comixed with rHuPH20 2000 U/mL by a pharmacist at the study site.

Patients in cohort 1 received SC atezolizumab 1800 mg injected in the thigh for 1 cycle. In cohort 2, patients received SC atezolizumab 1200 mg injected in the thigh every 2 weeks for 3 cycles, and in cohort 3, SC atezolizumab 1800 mg was administered every 3 weeks for 3 cycles in the abdomen for the first dose and then in the thigh for the remaining 2 doses. Following the SC regimen, patients in all cohorts received IV atezolizumab 1200 mg every 3 weeks for each subsequent cycle until disease progression, loss of clinical benefit, unacceptable toxicity, or withdrawal of consent (Figure S1).

PK samples of atezolizumab were collected from patients in cohort 1 during cycle 1 on day 1 before dosing and 8 hours after dosing; on days 2, 4, and 8; in cycles 2, 3, 4, 8, 12, and 16 on day 1 before dosing and within 30 minutes after infusion of IV atezolizumab; and at the visit at which treatment was discontinued. Timing of sample collection in cohorts 2 and 3 was similar, with the exception of cycles 2, 3, 8, 12, and 16, during which only a predose sample was collected on day 1, an additional sample was collected during cycle 5 on day 1 before dosing and 8 hours after dosing, and additional postadministration samples were collected during cycle 2 in cohort 3. PK samples were analyzed with validated assays at a central laboratory.

### Bioanalytical Methods

A validated sandwich enzyme‐linked immunosorbent assay wherein atezolizumab is captured with human programmed death‐ligand 1 and detected with an antiframework antibody was used to measure the concentration of atezolizumab in serum samples.^12^ The assay showed acceptable interassay precision (percent coefficient of variation [CV%]) and accuracy (percent difference) with ranges of 6.02% to 12.6% and 0.2% to 4.17%, respectively. The calibration range was 0.06 to 2.0 μg/mL, and the sensitivity limit was 0.06 μg/mL. In validation, the within‐ and between‐day variability (CV%) for the quality control samples at 5 concentrations was ≤4.12% and ≤4.59%.

### Pharmacokinetic Analyses: Noncompartmental Analysis and Model‐Based Evaluations to Determine the SC Dose of Atezolizumab

#### Noncompartmental Analysis

Individual and mean observed serum atezolizumab concentration vs time data were plotted by cohort. The PK of atezolizumab was characterized by estimating the cycle 1 PK parameters of C_trough_ at the end of cycle 1, area under the concentration‐time curve (AUC) from time 0 to day 21 (AUC_0‐21_; cohorts 1 and 3), AUC from time 0 to day 14 (AUC_0‐14_; cohort 2), maximum concentration (C_max_), and time to reach C_max_. Estimates for these parameters were tabulated and summarized (mean, standard deviation, geometric mean, geometric CV%, median, minimum, and maximum) by cohort.

#### Model‐Based Evaluations

To leverage the maximum of PK information observed in part 1, a population PK (popPK) analysis was conducted. Indeed, for this model‐based evaluation, all patients with at least 1 posttreatment PK observation could be used. In this analysis, the popPK model for atezolizumab administered IV^12^ was leveraged to fit part 1 PK data following both SC and IV administrations. Additional parameters were added to describe SC absorption, including the absorption rate constant (k_a_) and absolute bioavailability (F_1_). The impact of site of administration and other covariates suggested through exploratory graphical analysis were evaluated on those absorption parameters. Further details of the popPK analysis are reported in the Supplemental Information.

PK results were then compared with reference IV data from the OAK (NCT02008227)^2^ and IMpassion130 (NCT02425891)^5^ phase 3 studies of atezolizumab in previously treated locally advanced or metastatic NSCLC and previously untreated metastatic triple‐negative breast cancer, respectively.

Using the results of IMscin001 Part 1, model‐based simulations were performed to further support the dose selection for part 2 of the study. Four SC doses were tested in silico: 1600, 1800, 1875, and 2000 mg, each administered in the thigh on an every‐3‐week regimen. One thousand trials were simulated for each dose, mimicking a phase 3 study (part 2 of the IMscin001 study), to calculate the probability of providing drug exposures that are noninferior to IV atezolizumab 1200 mg every 3 weeks in terms of cycle 1 and steady‐state C_trough_ and AUC_0‐21_. For each simulated trial, the geometric mean ratio (GMR; SC/IV) for selected PK parameters (C_trough_ at cycle 1 [ie, cycle 2 before dosing], C_trough_ at steady state [ie, cycle 11 before dosing], and AUC_0‐21_ at cycle 1 and at steady state) was calculated. The percentage of trials with the lower bound of the 90% confidence interval of the GMR of >0.8 was tabulated to provide an estimated probability of success for each selected dose. The probabilities resulting from the simulations were used to support the selection of an SC atezolizumab dose that was likely to result in exposure not lower than that seen with IV atezolizumab in the proposed phase 3 (part 2) of this study. Further details of the PK simulations are reported in the Supplemental Information.

### Safety Analysis

All doses in part 1 were administered in a monitored setting, with patient assessment for toxicity performed before administration of the next dose. All patients who received ≥1 dose of SC atezolizumab or IV atezolizumab were included in the safety analysis. Safety was evaluated on the basis of the incidence and severity of AEs, assessment of left ventricular ejection fraction, and changes in clinical laboratory results. Serious AEs (SAEs), treatment‐related AEs, and AEs of special interest were documented. Summarized safety results include all AEs irrespective of route of administration, except when specified.

Investigators were responsible for evaluating the occurrence, severity, and seriousness of AEs and determination of the relationship to the study treatment. Treatment with SC or IV atezolizumab was discontinued if patients experienced intolerable toxicity determined to be related to study treatment and considered by the investigator to be unacceptable, given the individual patient's potential response to therapy and severity of the event. AEs occurring during or after study treatment administration considered to be related to study treatment infusion or injection were captured as a diagnosis (eg, “infusion‐related reaction” or “injection‐related reaction”) on the AE form. Following discontinuation of treatment, patients had a treatment discontinuation visit within 30 days before entering a 90‐day follow‐up period.

## Results

### Study Population and Demographics

**Figure 1:**
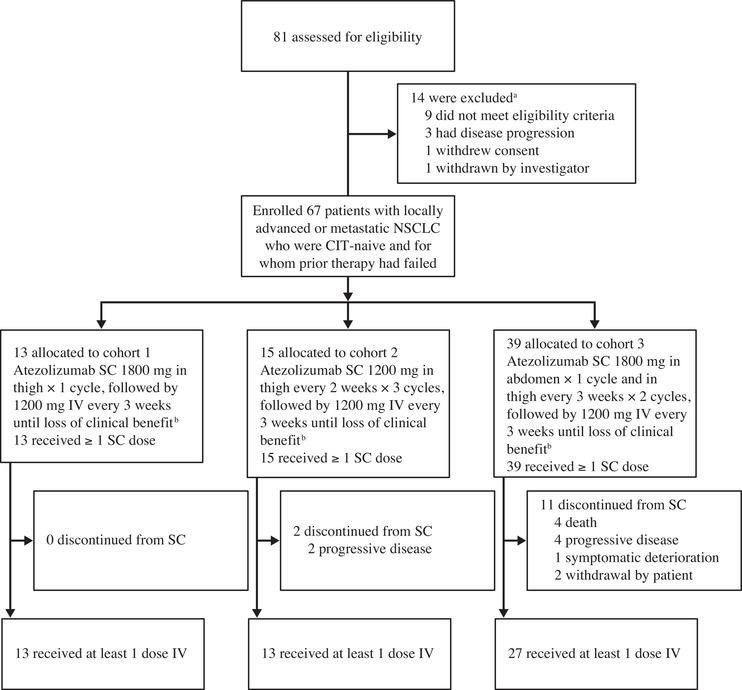
Trial profile. CIT, cancer immunotherapy; IV, intravenous; NSCLC, non–small cell lung cancer; SC, subcutaneous. ^a^Reason for study exclusion [number]: did not meet eligibility criteria [9] (inadequate hematologic and end‐organ function as determined by laboratory results within 14 days before initiation of study treatment [2]; body mass index too low [2]; treatment with systemic immunosuppressive within 2 weeks before enrollment [2]; severe infection within 4 weeks before initiation of study treatment [1]; Eastern Cooperative Oncology Group performance status >1 [1]; hepatitis core antibody B positive [1]); disease progression [3] (symptomatic, untreated, or actively progressing central nervous system metastases [2]; disease progression or recurrence after treatment with a platinum‐containing regimen for NSCLC [1]); withdrawal [1] (withdrew consent during screening [1]); withdrawn by investigator [1] (inability to comply with study protocol, investigator's judgment [1]). ^b^Cohort 1: one single dose of atezolizumab SC (in the thigh). Cohort 2: 3 cycles of atezolizumab SC (in the thigh). Cohort 3: 3 cycles of atezolizumab SC (first injection in the abdomen and subsequent injections in the thigh). Loss of clinical benefit includes progressive disease and pseudo‐progression.

Phase 1b of IMscin001 was conducted at 19 centers worldwide. Enrollment began December 27, 2018; the data cutoff date for dose selection in part 1 was March 10, 2020. A total of 67 patients (intent‐to‐treat population) was enrolled in part 1: 13 patients in cohort 1, 15 in cohort 2, and 39 in cohort 3 (Figure 1). All patients were evaluable for safety (safety‐evaluable population). Mean age was 64.2 years (range, 31‐83 years), with 41 (61.2%) men and 55 (82.1%) White patients. Nearly two‐thirds (44 [65.7%]) were classified with an Eastern Cooperative Oncology Group performance status of 1, 64 (95.5%) had metastatic disease, 6 (9.0%) had brain metastases, and 10 (14.9%) had liver metastases (Table 1). Mean treatment duration (standard deviation) of SC atezolizumab was 22.0 days (0.0) for cohort 1, 45.1 days (15.3) for cohort 2, and 56.1 days (22.1) for cohort 3.

**Table 1:** Baseline Demographics

	Cohort 1 Atezolizumab Comix 1800 mg SC (n = 13)	Cohort 2 Atezolizumab Comix 1200 mg SC (n = 15)	Cohort 3 Atezolizumab Comix 1800 mg SC (n = 39)	Total (N = 67)
**Age, y**				
n	13	15	39	67
Mean (SD)	62.7 (9.9)	62.9 (11.8)	65.2 (10.7)	64.2 (10.7)
Median	60.0	65.0	66.0	64.0
Min, max	49, 81	40, 82	31, 83	31, 83
**Age group, y**				
n	13	15	39	67
<65, n (%)	8 (61.5)	7 (46.7)	19 (48.7)	34 (50.7)
≥65, n (%)	5 (38.5)	8 (53.3)	20 (51.3)	33 (49.3)
**Sex**				
n	13	15	39	67
Male, n (%)	5 (38.5)	9 (60.0)	27 (69.2)	41 (61.2)
Female, n (%)	8 (61.5)	6 (40.0)	12 (30.8)	26 (38.8)
**Ethnicity**				
n	13	15	39	67
Hispanic or Latino, n (%)	1 (7.7)	5 (33.3)	12 (30.8)	18 (26.9)
Not Hispanic or Latino, n (%)	9 (69.2)	7 (46.7)	25 (64.1)	41 (61.2)
Not stated, n (%)	3 (23.1)	3 (20.0)	2 (5.1)	8 (11.9)
**Race**				
n	13	15	39	67
White, n (%)	8 (61.5)	11 (73.3)	36 (92.3)	55 (82.1)
Asian, n (%)	2 (15.4)	2 (13.3)	1 (2.6)	5 (7.5)
Unknown, n (%)	3 (23.1)	2 (13.3)	2 (5.1)	7 (10.4)
**Weight at baseline, kg**				
n	12	15	38	65
Mean (SD)	66.35 (10.97)	72.43 (17.12)	72.54 (13.88)	71.37 (14.21)
Median	65.35	70.00	73.20	71.00
Min, max	49.0, 89.0	52.0, 112.8	43.6, 94.0	43.6, 112.8
**Height at baseline, cm**				
n	12	15	38	65
Mean (SD)	165.41 (7.18)	166.01 (8.72)	167.07 (8.72)	166.52 (8.36)
Median	168.05	168.00	168.00	168.00
Min, max	155.0, 175.0	148.0, 180.0	150.0, 187.0	148.0, 187.0
**Body mass index at baseline, kg/m^2^**				
n	12	15	38	65
Mean (SD)	24.08 (3.23)	26.07 (4.73)	25.87 (4.02)	25.58 (4.07)
Median	23.00	26.00	26.00	26.00
Min, max	19.0, 29.0	18.0, 37.0	18.0, 32.0	18.0, 37.0
**ECOG PS**				
n	13	15	39	67
0, n (%)	3 (23.1)	7 (46.7)	13 (33.3)	23 (34.3)
1, n (%)	10 (76.9)	8 (53.3)	26 (66.7)	44 (65.7)
**Metastatic disease**				
n	13	15	39	67
Yes, n (%)	13 (100)	15 (100)	36 (92.3)	64 (95.5)
NA (no/unknown), n (%)	0	0	3 (7.7)	3 (4.5)
**Brain metastasis**				
n	13	15	39	67
Yes, n (%)	0	1 (6.7)	5 (12.8)	23 (34.3)
NA (no/unknown), n (%)	13 (100)	14 (93.3)	34 (87.2)	61 (91.0)
**Liver metastasis**				
n	13	15	39	67
Yes, n (%)	1 (7.7)	1 (6.7)	8 (20.5)	10 (14.9)
NA (no/unknown), n (%)	12 (92.3)	14 (93.3)	31 (79.5)	57 (85.1)

ECOG PS, Eastern Cooperative Oncology Group performance status; max, maximum; min, minimum; NA, not applicable; SC, subcutaneous.

Patients are grouped per treatment assigned. Data cutoff: March 10, 2020.

### Subcutaneous Atezolizumab PK: Noncompartmental Analysis

**Figure 2:**
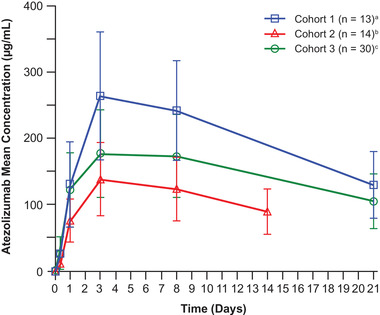
Serum atezolizumab cycle 1 concentration vs time profile by cohort (mean ± SD). SC, subcutaneous. ^a^Cohort 1: 1800 mg of SC atezolizumab (in the thigh). ^b^Cohort 2: 1200 mg of SC atezolizumab (in the thigh). ^c^ Cohort 3: 1800 mg of SC atezolizumab (in the abdomen).

Of the 67 patients with available PK data, data from 57 patients were used for noncompartmental analyses. Ten patients (1 [6.7%] in cohorts 2 and 9 [23.1%] in cohort 3) were excluded from the noncompartmental analyses because of an incomplete PK profile (5 patients), cycle 1 C_trough_ taken >2 days outside the planned collection day (4 patients), or duplicate time of collection (1 patient). Following administration of SC atezolizumab during cycle 1, C_trough_ was 121 μg/mL in cohort 1 (1800 mg every 3 weeks, thigh), 83.2 μg/mL in cohort 2 (1200 mg every 2 weeks, thigh), 97.3 μg/mL in cohort 3 (1800 mg every 3 weeks, abdomen). Mean serum SC atezolizumab concentrations over time for cycle 1 are shown in Figure 2.

### Subcutaneous Atezolizumab PK: Model‐Based Evaluation

A popPK analysis was performed on the data from 67 patients obtained in part 1 after both SC and IV administration. This approach enabled a robust estimation of the atezolizumab SC absorption parameters. The popPK model developed with IV atezolizumab PK data^12^ was used as a basis, and was extended, with the addition of a first‐order k_a_ and F_1_. The resulting popPK model for atezolizumab SC is reported in Table S1. Absorption parameters k_a_ for thigh and F_1_ for thigh and abdomen were estimated with high precision (relative SE of ≤ 8%). Bioavailability was estimated to be 82.9% for injection in the thigh and 71.1% for injection in the abdomen, with a large interindividual variability of 124%. Individual estimates of bioavailability ranged from 36.6% to 97.7% for the thigh and 29.7% to 95.5% for the abdomen. Of a total of 67 patients, 1 patient (1.5%) across cohorts had an individual estimate of F_1_ of <30.0%. Good modeling performance was shown by prediction‐corrected visual predictive check plots, observed PK profiles in general (Figure S2), and C_trough_ samples in particular (Figure S3) after SC administration were well within the prediction intervals of the popPK model.

Exposure following SC injection in the abdomen (cohort 3) was found to be lower than that observed in the thigh (cohort 1). Specifically, cycle 1 PK parameters for C_trough_, C_max_, and AUC_0‐21_ were 20%, 28%, and 27% lower, respectively, following injection of atezolizumab SC 1800 mg in the abdomen (cohort 3) than in the thigh (cohort 1) (Table 2). Following doses of 1800 mg in cohorts 1 and 3, and 1200 mg in cohort 2, serum concentrations of SC atezolizumab exhibited a dose‐dependent increase in exposure. In each cohort, time to reach C_max_ was reached within 3 to 4 days.

**Table 2:** Atezolizumab SC PK Results (Cycle 1)

Part 1: CIT‐Naive Patients With NSCLC That Progressed on Platinum‐Based Chemotherapy (N = 67)			
Cycle 1 PK Parameters	Cohort 1 Atezolizumab SC 1800 mg (thigh) (n = 13)	Cohort 2 Atezolizumab SC 1200 mg (thigh) (n = 14)	Cohort 3 Atezolizumab SC 1800 mg (abdomen) (n = 30)
C_trough_ GM, μg/mL (%CV)	121 (42.8)	83.2 (43.1)	97.3 (43.0)
Mean, μg/mL (SD)	130 (49.9)	89.6 (33.8)	105 (40.9)
C_max_ GM, μg/mL (%CV)	251 (40.9)	129 (42.5)	181 (38.3)
Mean, μg/mL (SD)	268 (97.4)	139 (54.4)	192 (63.3)
t_max_, d, median (min, max)	3.02 (2.93, 7.80)	3.45 (3.00, 8.95)	3.92 (2.99, 7.11)
AUC GM, μg ∙ d/mL (%CV)	3870 (38.6)	1410 (41.8)	2820 (38.6)
Mean, μg ∙ d/mL (SD)	4100 (1340) AUC_0‐21_	1520 (564) AUC_0‐14_	2990 (974) AUC_0‐21_

AUC, area under the concentration‐time curve; CIT, cancer immunotherapy; C_max_, maximum serum concentration; C_trough_, serum trough concentration; d, days; GM (%CV), geometric mean (percent coefficient of variation); max, maximum; min, minimum; NSCLC, non–small cell lung cancer; PK, pharmacokinetic; SC, subcutaneous; SD, standard deviation; t_max_, time to maximum serum concentration.

Mean data are log‐transformed.

Following a dose of IV atezolizumab 1200 mg every 3 weeks in the OAK trial,^2^ the observed cycle 1 C_trough_ was 76.0 μg/mL. In this phase 1b SC study, C_trough_ was 121.1 μg/mL for cohort 1 (atezolizumab 1800 mg, thigh) and 94.3 μg/mL for cohort 3 (atezolizumab 1800 mg every 3 weeks, abdomen). Furthermore, in OAK, the model‐predicted GM for cycle 1 AUC was 2978 μg ∙ d/mL, whereas the AUCs for part 1 of this study were 3868 μg ∙ d/mL in cohort 1 and 2824 μg ∙ d/mL in cohort 3.

Compared with model‐predicted day 14 IV atezolizumab data from the IMpassion130 trial in patients with untreated metastatic triple‐negative breast cancer,^5^ cycle 1 C_trough_ PK parameters observed in cohort 2 (thigh only) were similar (83.2 and 85.1 μg/mL, respectively). The median cycle 1 AUC_0‐14_ of cohort 2 was numerically lower than the model‐predicted one in IMpassion130 (1410 versus 1914 μg ∙ d/mL) but with substantial overlap.

Out of 822 atezolizumab concentration observations collected in 67 patients, only 12 values were found below the target concentration of 6 μg/mL (selected as the predicted target concentration assumed to provide 95% tumor receptor saturation needed for efficacy, based on preclinical evidence^13^), and none of those concentrations corresponded to C_trough_ samples at any cycle. Nine of these samples were collected at the cycle 1, day 1, at the 8‐hour time point, at which low concentrations are expected at the beginning of the absorption phase. The 3 other samples were collected 35 days (1 sample) and 99 days (2 samples) after the latest administered dose.

Model‐based simulations were performed to support the dose selection for part 2 (phase 3) of IMscin001. For administration in the thigh, the 1800‐mg every‐3‐week dose provided a high probability of exposure not lower than with 1200‐mg IV administration using all the selected exposure metrics (Table 3). For administration in the abdomen, sufficiently close exposures were not guaranteed, even with a higher dose of 2000 mg SC, especially for the cycle 1 AUC_0‐21_ parameter.

**Table 3:** Probabilities of Phase 3 Outcomes and Geometric Mean (%CV [90%CI]) Exposure Metrics From Clinical Trial Simulations With SC Administration Every 3 Weeks in the Thigh

		Atezolizumab Dose				
		SC, mg				IV, mg
Parameter		1600	1800	1875	2000	1200
Cycle 1 C_trough_, μg/mL	Probability, % GM (%CV) [90%CI]	100 88.1 (52.5) [37.6‐177]	100 99.3 (52.3) [42.4‐199]	100 103 (52.3) [44.2‐207]	100 110 (52.2) [47.1‐221]	NV 76.2 (42.7) [38.6‐141]
Cycle 1 AUC_0‐21d_, μg ∙ d/mL	Probability, % GM (%CV) [90%CI]	67.2 2625 (39.8) [1322‐4400]	99.0 2953 (39.8) [1487‐4950]	99.9 3076 (39.8) [1549‐5156]	100 3281 (39.8) [1652‐5500]	NV 2990 (23.5) [2096‐4403]
C_trough, SS_, μg/mL	Probability, % GM (%CV) [90%CI]	100 194 (65.5) [69.9‐473]	100 218 (65.5) [78.6‐532]	100 227 (65.5) [81.8‐555]	100 243 (65.5) [87.3‐592]	NV 169 (61.3) [65.9‐399]
AUC_0‐21d, SS_, μg ∙ d/mL	Probability, % GM (%CV) [90%CI]	95.0 5810 (50.2) [2589‐1648]	100 6536 (50.2) [2912‐13 103]	100 6808 (50.2) [3034‐13 649]	100 7262 (50.2) [3237‐14 558]	NV 5823 (38) [3237‐10 523]
Cycle 1 C_trough_, <6 μg/mL,^a^ %	…	0.0907	0.0697	0.0630	0.0525	0.0134%

AUC, area under the concentration‐time curve; C_max_, maximum serum concentration; C_trough_, serum trough concentration; GM (%CV), geometric mean (percent coefficient of variation); IV, intravenous; NV, no value; PK, pharmacokinetic; SC, subcutaneous; SS, steady state.

^a^ Percent of cycle 1 C_trough_ <6 μg/mL.

A dose of SC atezolizumab 1800 mg (from a concentration in the vial of 125 mg/mL) corresponds to 14.4 mL. Considering the limitations of syringe markings (1‐mL graduations on a 20‐mL syringe), SC atezolizumab is expected to be administered at a volume of 15 mL, corresponding to a dose of 1875 mg. The probabilities for phase 3 trial success with 1875 mg SC based on the lower bound of the 90% confidence interval of the GMR and the summary of simulated exposure metrics are also provided in Table 3.

**Figure 3:**
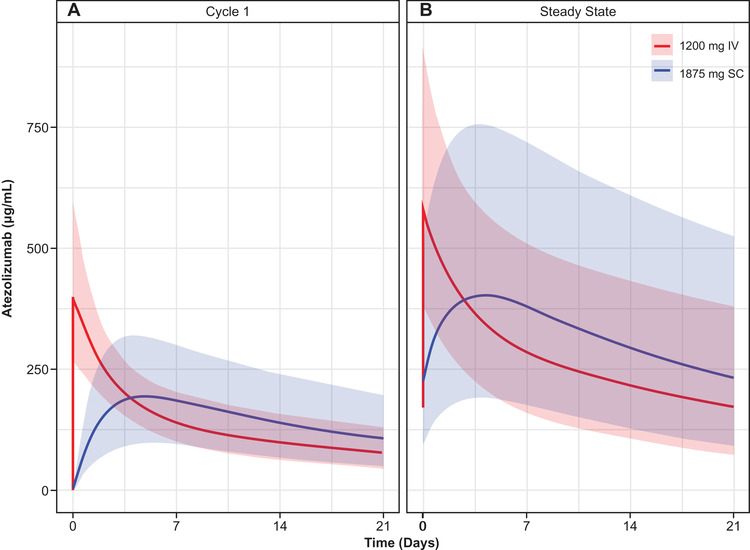
Simulated pharmacokinetic profiles of atezolizumab 1875 mg SC administration in the thigh in (A) cycle 1 and (B) at steady state, overlaid with 1200‐mg every‐3‐week IV administration. Solid lines are the medians of geometric means for each simulated dose; shaded areas are the 5th and 95th percentiles interval of geometric means for each simulated dose. IV, intravenous; SC, subcutaneous.

As expected, the simulated exposures after the administration of an SC dose of 1875 mg are close to those observed after the administration of the 1800‐mg dose. Therefore, the safety profile is expected to be similar. The proportion of cycle 1 C_trough_ samples expected to be <6 μg/mL following 1875‐mg every‐3‐week SC dosing in the thigh is very low (0.063%). Figure 3 shows the simulated typical PK profiles from SC administration at the selected dose of 1875 mg every 3 weeks in the thigh, overlaid with the corresponding simulations for 1200 mg IV.

### Safety

Atezolizumab SC was well tolerated, and the safety profile was consistent with the known risks of atezolizumab IV.^1^, ^2^, ^3^, ^4^, ^5^, ^6^, ^7^, ^8^, ^9^, ^10^ Safety was comparable across cohorts, and no clinically significant differences in the safety profile between cohorts were identified. No new safety signals were observed. Safety results are summarized in Table 4.

**Table 4:** Safety Summary

	Cohort 1 Atezolizumab SC 1800 mg (thigh) (n = 13)	Cohort 2 Atezolizumab SC 1200 mg (thigh) (n = 15)	Cohort 3 Atezolizumab SC 1800 mg (abdomen, then thigh) (n = 39)
No. of patients (%) with ≥1 AE	13 (100)	13 (86.7)	31 (79.5)
No. of AEs	84	111	198
No. (%) of patients with ≥1:			
Treatment‐related AE	11 (84.6)	8 (53.3)	25 (64.1)
Grade 3/4 AE	1 (7.7)	4 (26.7)	9 (23.1)
Treatment‐related grade 3/4 AE	1 (7.7)	2 (13.3)	5 (12.8)
Grade 5 AE	1 (7.7)	1 (6.7)	2 (5.1)
Treatment‐related grade 5 AE	0	0	0
Serious AE	2 (15.4)	4 (26.7)	9 (23.1)
Treatment‐related serious AE	0	1 (6.7)	3 (7.7)
AE leading to discontinuation of atezolizumab	0	1 (6.7)	2 (5.1)
AE leading to interruption of atezolizumab	3 (23.1)	3 (20.0)	4 (10.3)

AE, adverse event; IV, intravenous; SC, subcutaneous.

All patients who received ≥1 dose of SC atezolizumab or IV atezolizumab were included in the safety analysis. Investigator text for AE encoded using Medical Dictionary for Regulatory Activities, version 22.1. Percentages are based on N in the column headings. AEs collected after first treatment dose are included. Multiple occurrences of the same AE in 1 individual are counted only once except for the row labeled “No. of AEs,” in which multiple occurrences of the same AE were counted separately.

Overall, 57 patients (85.1%) had ≥1 AE, including 14 (20.9%) who experienced grade 3/4 and 4 (6.0%) who experienced grade 5 (fatal) events. Forty‐four patients (65.7%) experienced ≥1 treatment‐related AE, of which 8 (11.9%) were grade 3/4. No grade 5 treatment‐related AEs were reported. The most common AEs (≥ 15% of total) were anemia, asthenia, cough, fatigue, and nausea. Among cohorts (Table 4), the proportions of patients with grade 3/4 AEs included 1 patient (7.7%) in cohort 1, 4 patients (26.7%) in cohort 2, and 9 patients (23.1%) in cohort 3. Two patients (15.4%) in cohort 1 experienced SAEs, whereas the occurrence of SAEs in cohort 2 (4 patients [26.7%]) and cohort 3 (9 patients [23.1%]) were comparable. SAEs reported in ≥2 patients (3.0% of all patients) included pneumonia (1 in cohort 1 and 1 in cohort 3).

Reported grade 5 AEs were cerebrovascular accident (1 patient [7.7%] in cohort 1), pulmonary embolism (1 patient [6.7%] in cohort 2), unexplained death, and ischemic stroke (2 patients [5.1%] in cohort 3). One of the 4 cases (unexplained death) occurred during SC administration. All of these AEs were assessed by the investigator as unrelated to the study treatment. The 3 patients with cerebrovascular accident, pulmonary embolism, and ischemic stroke experienced their respective events during IV infusion, and each had preexisting risk factors according to their medical histories. There is limited information about the patient who experienced the event reported as death during SC administration because hospitalization was at a nonstudy site.

Across all cohorts, the majority of AEs of special interest (21 of 26 [80.8%]) did not exceed grade 1 or 2 in severity. There were no reports of infusion‐related reactions or anaphylaxis in any of the cohorts. The events observed were consistent with the known profile of atezolizumab.^1^, ^2^, ^3^, ^4^, ^5^, ^6^, ^7^, ^8^, ^9^, ^10^ Similarly, the majority of AEs occurring during cycle 1 (SC administration only) were of grade 1/2 in severity (71 of 77 [92.2%]), indicating no safety signal from the SC atezolizumab or site of administration. Seven of 11 patients experienced an injection site reaction in cycle 1. Patients with injection‐site reactions included 4 patients (30.8%) in cohort 1, 2 patients (13.3%) in cohort 2, and 5 patients (12.8%) in cohort 3. Ten of 11 (90.9%) injection‐site reactions were grade 1; 1 patient (6.7%) in cohort 2 experienced a grade 2 injection‐site reaction that resolved without sequelae. The most common terms included reaction at the injection site, injection site pain, and injection site erythema. AEs specific to the SC injection site and surrounding skin area appeared to be generally low grade and well tolerated (grade 1, 16/17 [94.1%], and grade 2, 1/17 [5.9%]).

Three patients in the safety‐evaluable population (1 [6.7%, pulmonary embolism] in cohort 2, and 2 [5.1%, grade 2, bronchitis; grade 5, unexplained death] in cohort 3) discontinued treatment with atezolizumab because of an AE. Only 1 AE (1.5% [grade 5, unexplained death]) in cohort 3 led to SC atezolizumab discontinuation, with the remaining discontinuations occurring during IV administration. These AEs were considered unrelated to study treatment by the investigator.

## Discussion

SC formulations can provide additional options for safe administration of monoclonal antibodies beyond the hospital setting, including the home.^24^, ^27^ Patient and practitioner preferences for SC administration compared with IV infusion are linked with spending less time in the clinic, easier administration, improved workflow, and scheduling flexibility; additional options for patients with fluid restrictions or difficult venous access; and potential for cost savings.^18^, ^19^, ^20^, ^21^, ^22^, ^23^, ^24^, ^25^, ^26^, ^27^, ^28^, ^29^, ^30^, ^31^, ^32^, ^33^, ^34^ The benefits of SC administration become particularly relevant when external factors (eg, a pandemic, such as severe acute respiratory syndrome coronavirus 2 infection) disrupt and limit usual clinic work flow and increase the risk of exposure to life‐threatening clinical conditions to patients with cancer. Minimizing contact time for these vulnerable patients in hospital and clinic settings can help reduce the risk of virus exposure and transmission, which, if infection occurs, may lead to poorer outcomes due to compromised immune systems.^46^, ^47^, ^48^

IMscin001 is a global phase 1b/3 study in cancer immunotherapy–naive patients with locally advanced or metastatic NSCLC that has progressed on platinum‐based chemotherapy. The main aim of phase 1b was to identify the dose of SC atezolizumab that would yield comparable exposure to IV atezolizumab on the basis of serum C_trough_ at cycle 1.

Atezolizumab PK data after both SC and IV administrations were modeled using a population PK approach. A higher bioavailability was estimated for administration in the thigh (82.9%) compared with the abdomen (71.1%), with large overlap of individual values given the large interindividual variability.

In part 1 of IMscin001, SC atezolizumab was administered as monotherapy. Doses were 1800 mg (1 dose in the thigh), 1200 mg every 2 weeks (3 doses in the thigh), or 1800 mg every 3 weeks (first dose in the abdomen and the following 2 doses in the thigh), and in each cohort followed by IV atezolizumab 1200 mg every 3 weeks. SC atezolizumab comix, given at a dose of 1800 mg every 3 weeks in the thigh, provided higher observed cycle 1 C_trough_ and AUC_0‐21_ values than IV atezolizumab given in the OAK study at a dose of 1200 mg every 3 weeks (cycle 1 C_trough_ [CV%]). Furthermore, significant overlap with values in OAK was observed: 121.1 μg/mL (42.8%) vs 76.0 μg/mL (53.9%), respectively; cycle 1 AUC_0‐21_: 3870 μg ∙ d/mL (38.6%) vs 2978 μg ∙ d/mL (26.1%), respectively.

SC atezolizumab was well tolerated and exhibited a safety profile consistent with the established safety profile of the IV formulation. No new or significant safety concerns were identified, and no differences between cohorts were observed. Injection‐site reactions were low grade and well tolerated.

Simulations of phase 3 using the popPK model indicated that an SC dose of 1875 mg every 3 weeks injected in the thigh has a high probability of providing drug exposures that are not lower than those seen with IV atezolizumab 1200 mg every 3 weeks in terms of cycle 1 and steady‐state C_trough_ and AUC_0‐21_.

## Conclusions

In this phase 1b dose‐finding study, administration of SC atezolizumab appeared to be feasible and well tolerated. Therefore, development of SC atezolizumab can be advanced to part 2 of IMscin001, a phase 3 study to confirm that the observed atezolizumab exposure following SC administration is sufficiently close to that seen with IV administration.

## Conflicts of Interest

L.A.H.‐B. and E.R. are employees of and hold stock in F. Hoffmann‐La Roche. P.C. is an employee of Genentech/Roche and holds stock in Roche Holding Ltd. S.K. is an employee of ProUnlimited. V.M., P.C., and B.W. are employees of Genentech, Inc. E.P., M.M., and N.T. are employees of F. Hoffmann‐La Roche Ltd. M.M. is an employee of Certara and has received consulting fees from Genentech, Inc., in connection with this work. K.T. is an employee of Chugai Pharmaceutical Co., Ltd., and has received financial support from Genentech, Inc. All authors received support for third‐party writing assistance for this manuscript, provided by F. Hoffmann‐La Roche Ltd.

No authors have disclosed they are Fellows of the American College of Clinical Pharmacology.

## Funding

This study was sponsored by F. Hoffmann‐La Roche Ltd, Basel, Switzerland. Support for third‐party writing assistance for this manuscript, furnished by M. Evelyn Rose, PharmD, of Health Interactions, Inc, was provided by F. Hoffmann‐La Roche Ltd.

## Data‐Sharing Statement

Qualified researchers may request access to individual patient‐level data through the clinical study data request platform (https://vivli.org/). Further details on Roche's criteria for eligible studies are available (https://vivli.org/members/ourmembers/). For further details on Roche's Global Policy on the Sharing of Clinical Information and how to request access to related clinical study documents, see https://www.roche.com/research_and_development/who_we_are_how_we_work/clinical_trials/our_commitment_to_data_sharing.htm.

## Supporting information

Supporting Information
