# Supplementary material for: Results of a Dose‐Finding Phase 1b Study of Subcutaneous Atezolizumab in Patients With Locally Advanced or Metastatic Non–Small Cell Lung Cancer
Source: Clin Pharmacol Drug Dev. 2021 Mar 31;10(10):1142–55. doi: 10.1002/cpdd.936 (PMC8518371; doi:10.1002/cpdd.936)
Supplement: Supplementary file 1 — Supporting Information [file CPDD-10-1142-s001.docx]

**Supplementary Materials: Results of a dose-finding phase 1b study of subcutaneous atezolizumab in patients with locally advanced or metastatic non-small cell lung cancer**

**Enriqueta F, et al.**

**Supplementary Methods**

Population Pharmacokinetic Modeling

Nonlinear mixed-effects modeling of the pharmacokinetic (PK) data was performed using a population approach with combined subcutaneous (SC) and intravenous (IV) data from Part 1 to characterize the absorption phase of the SC route of administration. The population PK (popPK) of atezolizumab after IV administration has been well described using a 2-compartment model with first-order elimination (Stroh, et al. 2017). This model, which was used for comparison, was parameterized in terms of clearance; volume of distribution of central compartment (V_2_); volume of distribution of peripheral compartment (V_3_); distributional clearance, with covariate effects of albumin, posttreatment antidrug antibody (ADA) status, tumor burden, and body weight on clearance, albumin, body weight, sex on V_2_, and sex on V_3_. Additional parameters were added to describe SC absorption, including the absorption rate constant (k_a_) and absolute bioavailability (F_1_). Site of administration and other covariates suggested through exploratory graphical analysis were tested on absorption parameters using univariate screening (descriptive *P* ≤ .05) and backward elimination (descriptive *P* ≤ .05). The model was evaluated using standard goodness-of-fit plots and prediction-corrected visual predictive checks (Bittner, et al. 2012). Individual exposure metrics (ie, C_trough_ and area under the concentration-time curve at cycle 1 and steady state [cycle 10]) were derived from the empirical Bayesian estimates of the popPK model parameters.

Dose Selection Simulations

Using the same study design as that proposed for the Part 2 segment of this study to compare various doses of SC atezolizumab with IV atezolizumab 1200 mg administered q3w, the popPK model was used to simulate 1000 phase 3 clinical trials. Four SC doses were tested: 1600 mg, 1800 mg, 1875 mg, and 2000 mg, each on a q3w regimen. Per label guidelines, the first simulated IV infusion was assumed to be given over 60 minutes and subsequent infusions over 30 minutes.

The study population covariate distributions and correlations were assumed to be similar to those of the OAK study (Rittmeyer, et al. 2017), which had a comparable patient population (ie, patients with locally advanced or metastatic non-small cell lung cancer who were cancer immunotherapy-naive and had progressed during or following a platinum-containing regimen). Sex and treatment-emergent ADA status were selected by random draw from 4 categories (male or female ADA negative and male or female ADA positive). Albumin values were assumed to be normally distributed with a mean of 38.5 g/L and SD of 5.76 g/L, with minimum and maximum boundaries of 3.2 g/L and 55 g/L, respectively. Different body weight distribution ranges with minimum and maximum boundaries were used for men and women. Baseline tumor burden values were stratified by treatment-emergent ADA status (mean of 4.13 and SD of 0.64 of the natural log of baseline tumor burden in millimeters, with minimum of 10 mm and maximum of 316 mm for ADA negative, and mean of 4.29 and SD of 0.7 of the natural log of baseline tumor burden in millimeters, with minimum of 13 mm and maximum of 287 mm for ADA positive).

Interindividual variability, residual variability, and uncertainty of the popPK model parameter estimates were also accounted for in the clinical trial simulations.

For each simulated trial, the geometric mean ratio (GMR; SC/IV) for selected PK parameters (C_trough_ at cycle 1 [ie, cycle 2 predose], C_trough_ at steady state [ie, cycle 11 predose], and AUC_0-21_ at cycle 1 and at steady state) was calculated. The percentage of trials with the lower bound of the 90% CI of the GMR of > 0.8 (non-inferiority margin) was tabulated to provide an estimated probability of success for each selected dose. The probabilities resulting from the simulations were used to support the selection of an SC atezolizumab dose that was likely to result in noninferior exposure to IV atezolizumab in the proposed phase 3 (Part 2) of this study.

**Supplementary References**

Bittner B, Richter WF, Hourcade-Potelleret F, et al. Development of a subcutaneous formulation for trastuzumab - nonclinical and clinical bridging approach to the approved intravenous dosing regimen. Arzneimittelforschung. 2012;62(9):401-409.

Rittmeyer A, Barlesi F, Waterkamp D, et al. Atezolizumab versus docetaxel in patients with previously treated non-small-cell lung cancer (OAK): a phase 3, open-label, multicentre randomised controlled trial. Lancet. 2017;389(10066):255-265.

Stroh M, Winter H, Marchand M, et al. Clinical Pharmacokinetics and Pharmacodynamics of Atezolizumab in Metastatic Urothelial Carcinoma. Clin Pharmacol Ther. 2017;102(2):305-312.

**Supplementary Figure S1.**


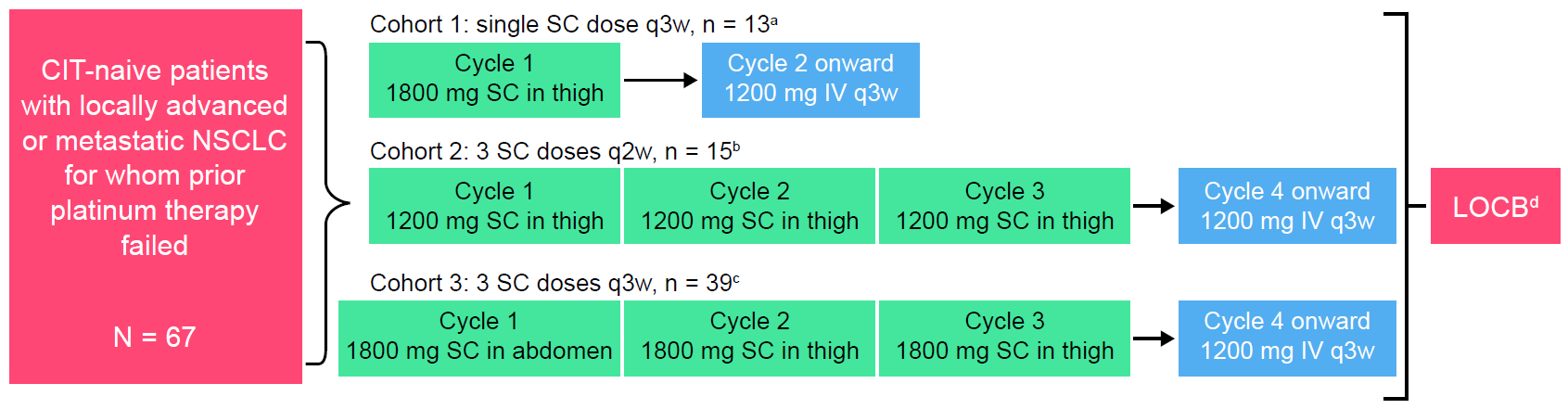


**Supplementary Figure S1.** IMscin001: Part 1 study design. SC atezolizumab refers to atezolizumab mixed with recombinant human hyaluronidase PH20. ^a^ Cohort 1: a single dose of SC atezolizumab (in the thigh). ^b^ Cohort 2: 3 cycles of SC atezolizumab (in the thigh). ^c^ Cohort 3: 3 cycles of SC atezolizumab (first injection in the abdomen and subsequent injections in the thigh). ^d^ Includes progressive disease and pseudoprogression. CIT, cancer immunotherapy; IV, intravenous; LOCB, loss of clinical benefit; NSCLC, non-small cell lung cancer; q2w, every 2 weeks; q3w, every 3 weeks; SC, subcutaneous.

**Supplementary Figure S2.**


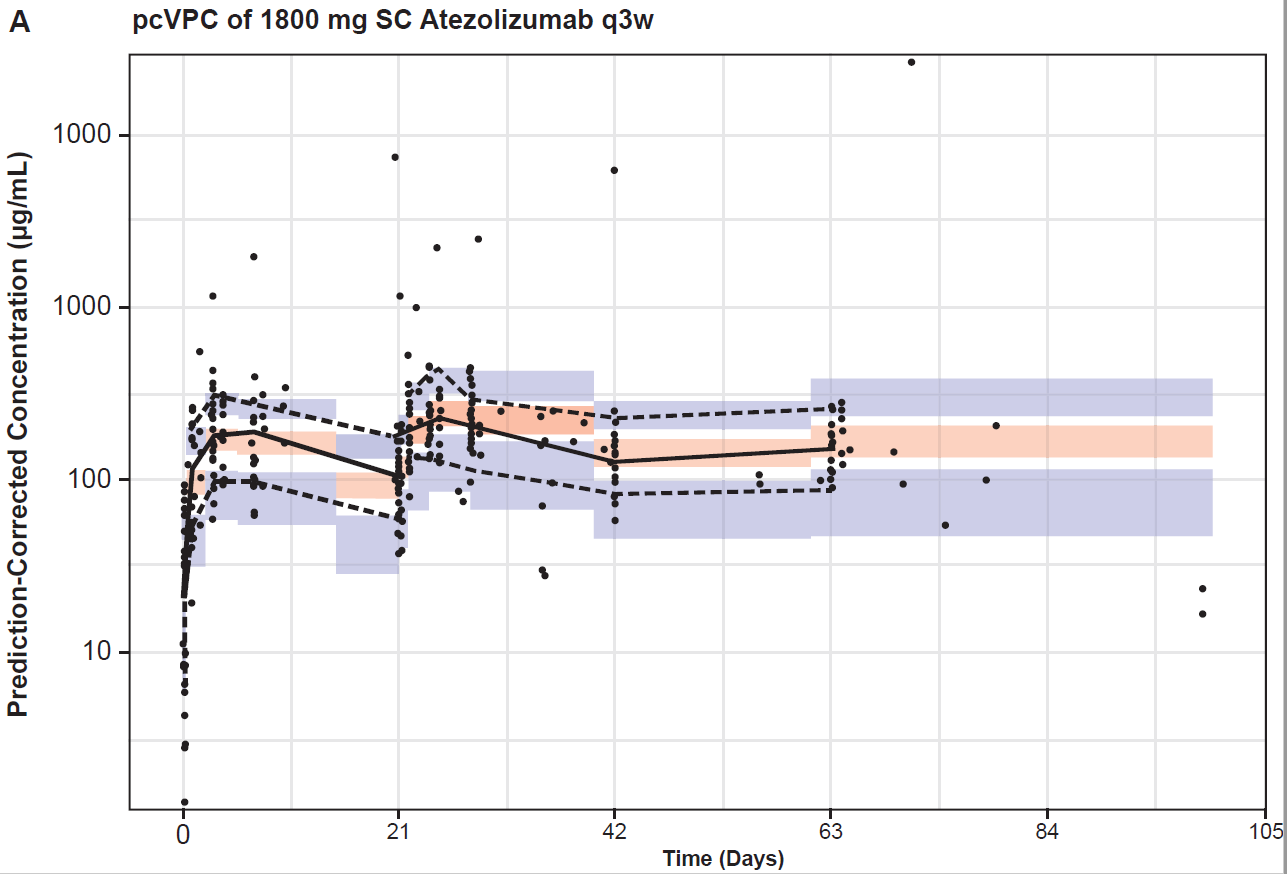


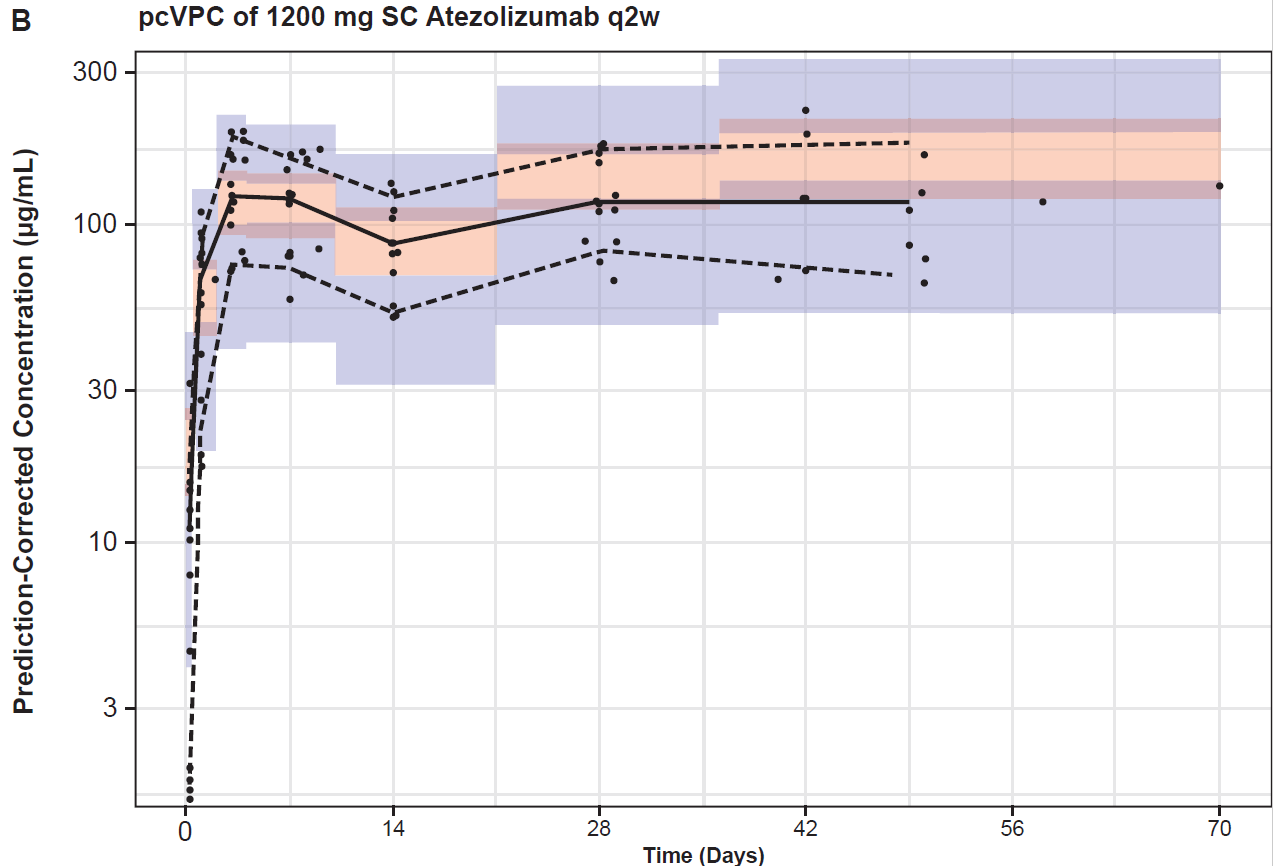


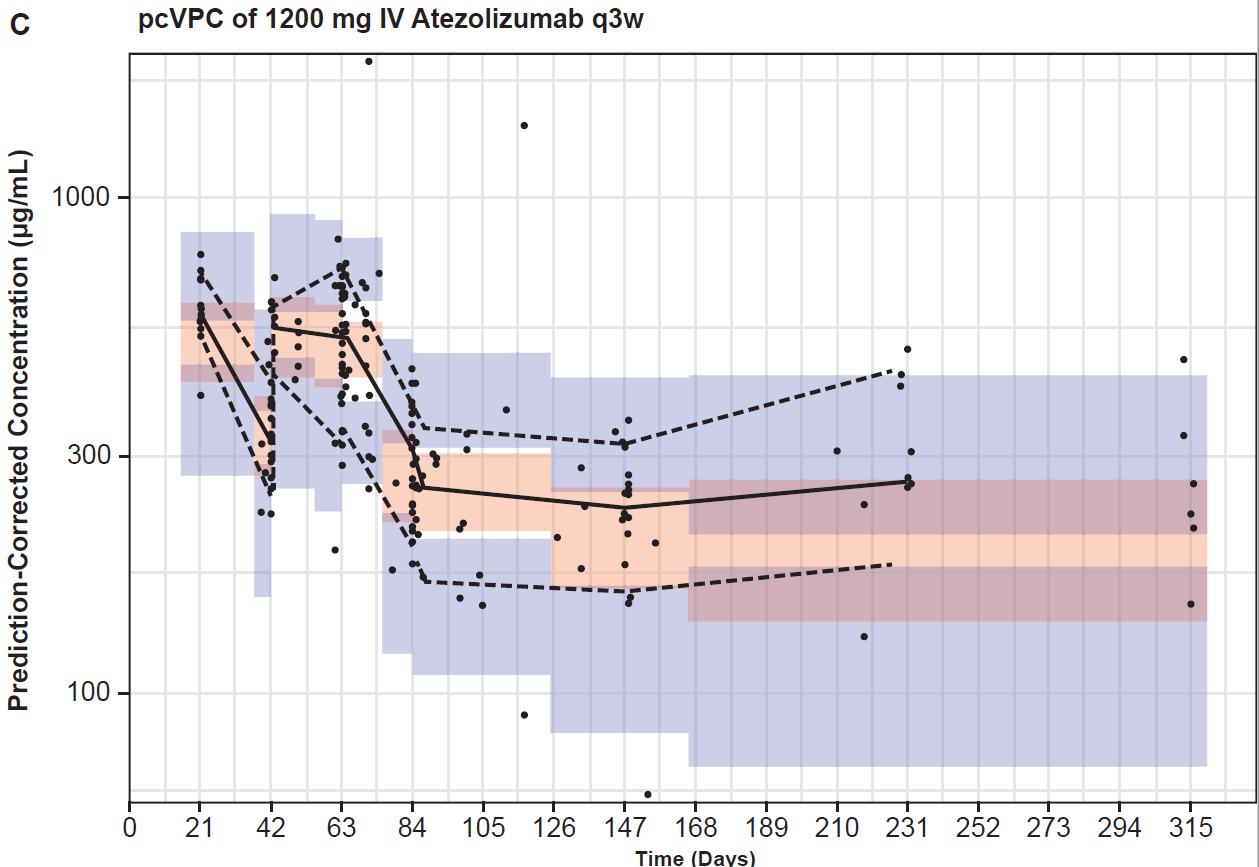


**Supplementary Figure S2.** Prediction-corrected visual predictive check plots of the PK profiles stratified by dose and regimen. (A) 1800 mg q3w SC; (B) 1200 mg q2w SC; and (C) 1200 mg q3w IV. Black dots are prediction-corrected observed data; solid black line is the 50th percentile; dashed black lines are the 10th (lower dashed line) and 90th (upper dashed line) percentiles of the prediction-corrected observed data. Pink-shaded areas represent the 95% CIs of the median; the areas shaded in blue represent the 95% CIs of the 10th and 90th percentiles, from simulations of 1000 replicates using the population PK model. IV, intravenous; PK, pharmacokinetics; pcVPC, prediction-corrected visual predictive check; q2w, every 2 weeks; q3w, every 3 weeks; SC, subcutaneous.

**Supplementary Figure S3.**


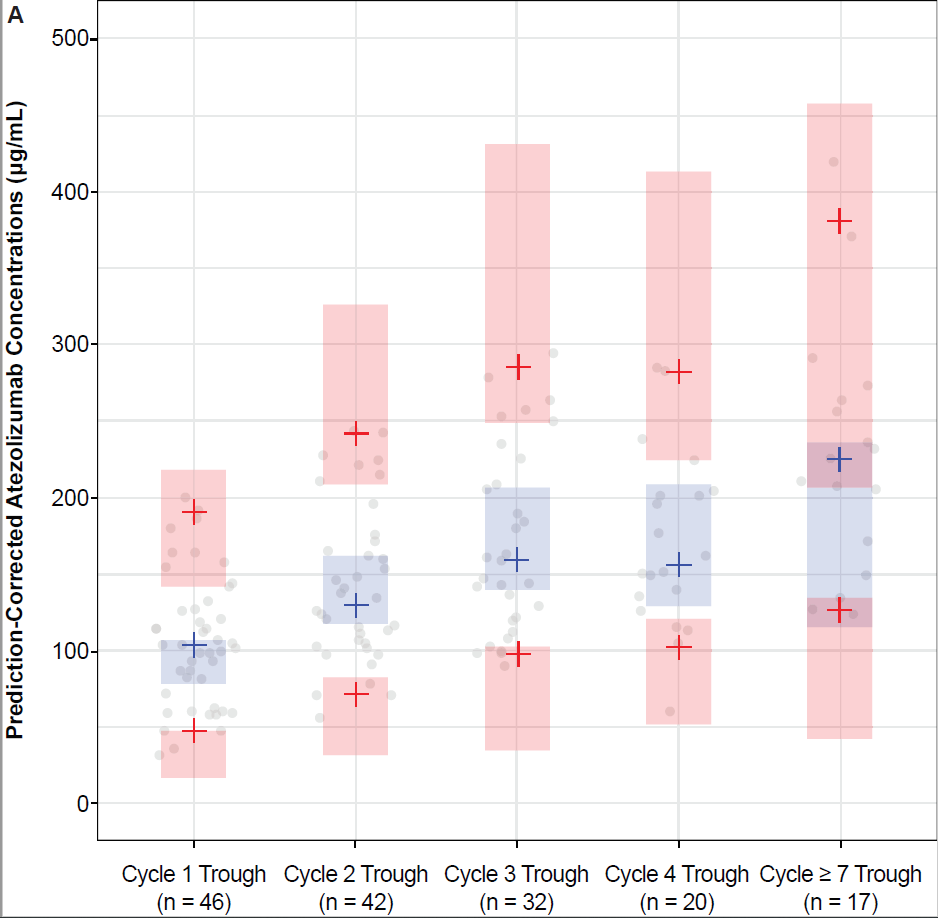


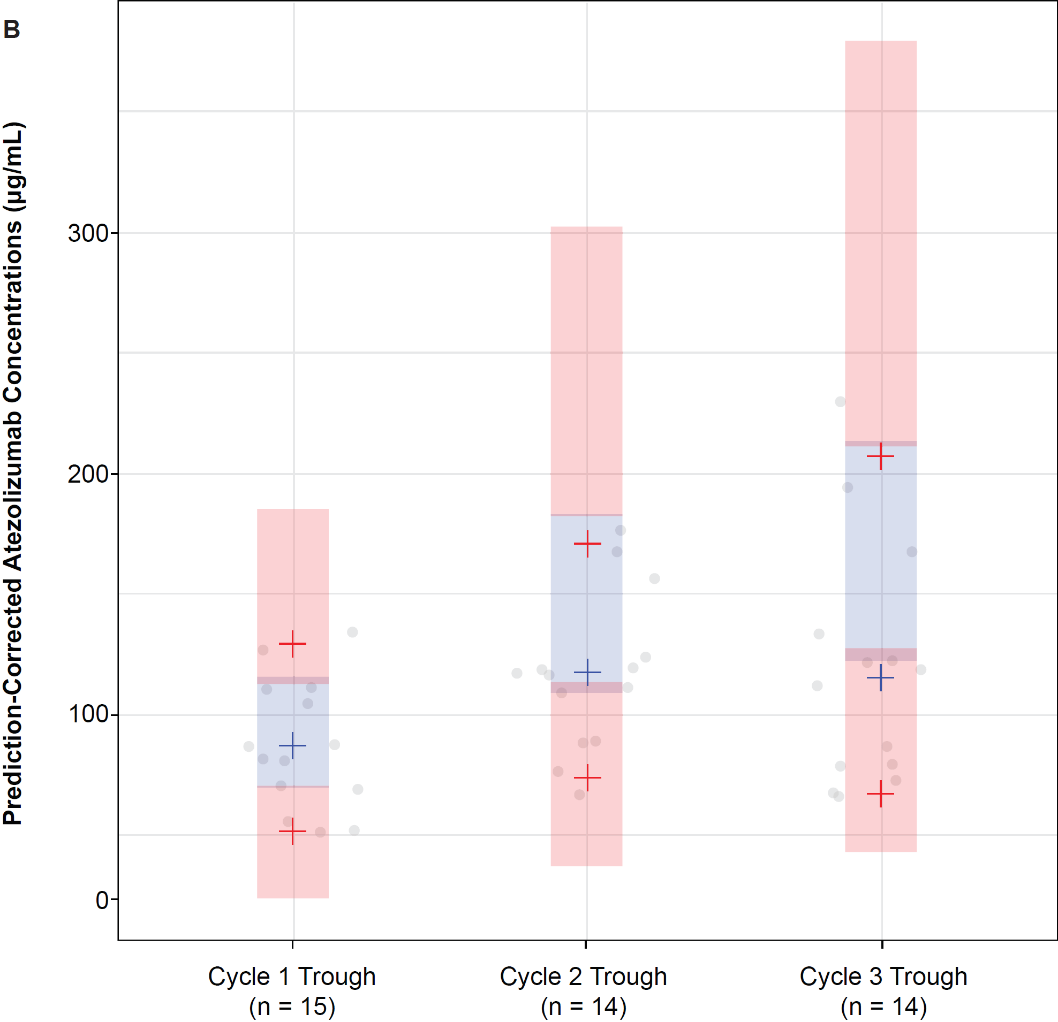


**Supplementary Figure S3.** Prediction-corrected visual predictive check plots of trough concentration data stratified by cohort. (A) Cohort 1, SC atezolizumab 1800 mg q3w and cohort 3, 1200 mg IV q3w. (B) Cohort 2, SC atezolizumab 1200 mg q2w. Only bins with ≥ 15 observations for cohorts 1 and 3, or with ≥ 10 observations for cohort 2, are shown. Gray dots are prediction-corrected observed data; blue and red crosses are 50th and 95th percentiles, respectively, of the prediction-corrected observed data. Blue shaded areas represent the 95% CIs of the median, and the pink shaded areas represent the 95% CIs of the 5th and 95th percentiles from simulations of 1000 replicates using the population pharmacokinetic model. IV, intravenous; q2w, every 2 weeks; q3w, every 3 weeks; SC, subcutaneous.

**Supplementary Table S1. Parameter Estimates of the popPK Model With Atezolizumab SC and IV Administration**

| **Parameter** | **Estimate** | **Parameter Fixed to PopPK Model Estimate (IV)** | **Relative SE** | **Shrinkage** |
| --- | --- | --- | --- | --- |
| Clearance, L/d | 0.2 | FIX |  |  |
| Volume of distribution, central compartment, L | 3.28 | FIX |  |  |
| Volume of distribution, peripheral compartment, L | 3.63 | FIX |  |  |
| Distributional clearance, L/d | 0.546 | FIX |  |  |
| Effect of baseline albumin on clearance | −1.12 | FIX |  |  |
| Effect of treatment-emergent ADA status on clearance | 0.159 | FIX |  |  |
| Effect of baseline tumor burden on clearance | 0.125 | FIX |  |  |
| Effect of baseline body weight on clearance | 0.808 | FIX |  |  |
| Effect of baseline albumin on the volume of distribution, central compartment | −0.350 | FIX |  |  |
| Effect of baseline body weight on the volume of distribution, central compartment | 0.559 | FIX |  |  |
| Effect of sex on the volume of distribution, central compartment | −0.129 | FIX |  |  |
| Effect of sex on the volume of distribution, peripheral compartment | −0.272 | FIX |  |  |
| **Absorption rate constant (1/d), thigh** | **0.269** |  | **5%** |  |
| **Bioavailability, thigh** | **0.829** |  | **5%** |  |
| **Absorption rate constant (1/d), abdomen** | **0.197** |  | **79%** |  |
| **Bioavailability, abdomen** | **0.711** |  | **8%** |  |
| **ω (on SD scale)—Interindividual Variability** | | | | |
| Interindividual variability, clearance | 29.4% | FIX |  |  |
| Interindividual variability, volume of distribution, central compartment | 18.1% | FIX |  |  |
| Interindividual variability, volume of distribution, peripheral compartment | 33.8% | FIX |  |  |
| Interindividual variability, clearance | 0% | FIX |  |  |
| **Interindividual variability, absorption rate constant** | **28.6%** |  | **65%** | **23%** |
| **Interindividual variability, volume of distribution, bioavailability** | **124%** |  | **17%** | **20%** |
| **σ (residual error)** | | | | |
| Proportional error | 0.0433 | FIX |  |  |
| Additive error | 16.6 | FIX |  |  |

ADA, antidrug antibody; IV, intravenous; popPK, population PK; SC, subcutaneous.

**Supplemental List: IMscin001 Study Centers**

*Chile*

Bradford Hill Centro de Investigación Clinica, Recoleta, Chile

*France*

Assistance Publique—Hôpitaux de Marseille, Marseille, France

Ico Rene Gauducheau, Oncologie, Saint Herblain, France

*Italy*

ASST Papa Giovanni XXIII, Oncologia Medica, Bergamo, Italy

IRCCS Istituto Clinico Humanitas, Oncologia, Rozzano, Italy

*Republic of Korea*

Asan Medical Center, Seoul, Republic of Korea

Samsung Medical Centre, Medical Oncology, Seoul, Republic of Korea

*Latvia*

Riga East Clinical University Hospital Latvian Oncology Centre, Riga, Latvia

*New Zealand*

Christchurch Clinical Studies Trust Ltd, Christchurch, New Zealand

*Poland*

Uniwersyteckie Centrum Kliniczne; Osrodek Badan Wczesnych Faz, Gdańsk, Poland

Regionalny Szpital Specjalistyczny im. W. Bieganskiego, Oddzial Onkologii Klinicznej, Grudziądz, Poland

Mazowieckie Centrum Leczenia Chorob Pluc i Gruzlicy; Department of Pulmonology and Subdivision of Oncology, Otwock, Poland

Narodowy Instytut Onkologii im. M. Sklodowskiej-Curie; Oddzial Badan Wczesnych Faz, Warszawa, Poland

*Spain*

Hospital Universitario Vall d’Hebron—PPDS, Barcelona, Spain

Hospital Universitario La Paz; Servicio de Oncología, Madrid, Spain

*United Kingdom*

Birmingham Heartlands Hospital, Birmingham, United Kingdom

St James Hospital; Department of Oncology/Hematology, Leeds, United Kingdom
